# The relationship between sense of coherence and emotional intelligence as individual health assets for mental health promotion in students and healthcare professionals: a scoping review

**DOI:** 10.3389/fpubh.2024.1304310

**Published:** 2024-02-20

**Authors:** Valentina Urtubia-Herrera, María Victoria Navarta-Sánchez, Ana María Palmar-Santos, Azucena Pedraz-Marcos, Alberto García-Gomez, Elkin O. Luis, Elena Bermejo-Martins

**Affiliations:** ^1^Medicine and Surgery Program, Doctoral School, Autonomous University of Madrid, Madrid, Spain; ^2^Nursing Department, Faculty of Medicine, Autonomous University of Madrid, Madrid, Spain; ^3^Nursing and Health Care Research Group, Puerta de Hierro-Segovia Arana Health Research Institute, Madrid, Spain; ^4^Health Care and Health Services Research Unit (Investén-ISCIII), Carlos III Health Institute (ISCIII), Madrid, Spain; ^5^Nursing Department, Pediatrics Unit, La Paz University Hospital, Madrid, Spain; ^6^Psychological Processes in Education and Health Group, School of Education and Psychology, University of Navarra, Pamplona, Spain; ^7^Navarra Institute for Health Research (IdiSNA), Pamplona, Spain; ^8^Department of Psychology, University of Copenhagen, Copenhagen, Denmark

**Keywords:** emotional intelligence, healthcare professionals, scoping review, sense of coherence, students

## Abstract

**Introduction:**

Workplace Mental health promotion in healthcare sector, is a global priority due to the stress associated with caregiving environments and the increase of mental health problems among health professionals and students. The role of emotional intelligence (EI) and sense of coherence (SOC) have been identified as critical health protectors. However, the relationship between them as well as the underlying mechanisms of these relationships on health benefits in this population is still unclear.

**Aim:**

To synthetize the existing literature on the relationship between emotional intelligence and sense of coherence, as well as their mutual impact on healthcare workers’ and student’s well-being.

**Method:**

A scoping review was conducted following the Joanna Briggs Institute guidelines. A systematic search was conducted in PsyCINFO, CINHAL, SCOPUS and PUBMED databases, using key-terms such as students, health professionals, emotional intelligence, and sense of coherence.

**Results:**

A total of 11 articles were included, with a range of years from 2014 to 2022. Evidence was found to support the positive relationship between sense of coherence and emotional intelligence. The use of EI as a training pathway to improve SOC and health promoting behaviors is suggested. The benefits of intervening on these factors contribute to improved health professionals’ and students’ general well-being and motivation for a better performance, either in their studies or clinical work.

**Conclusion:**

The positive relationship between emotional intelligence and a sense of coherence has direct and indirect benefits on students’ and healthcare professionals’ well-being. Future studies should address longitudinal and experimental analysis to confirm these findings.

## 1 Introduction

The World Health Organization (WHO) recognizes that the demands of the work environment can pose a risk to people’s health, making it a concern for governments, employers, and workers worldwide (1). In view of these implications, the protection and promotion of mental health is proposed as a global objective in the latest WHO Comprehensive Plan of Action for Mental Health 2013–2030 (2). This was the basis for the proposal of the Guidelines on Mental Health in the workplace, which include the development of positive aspects in the work environment, as well as the qualities and skills of workers (1).

Healthcare workers are exposed to stressful contexts, where working conditions and occupational hazards in the health sector have consequences on their health due to occupational diseases or accidents. These situations generate a significant cost for the health of the professional and also for the patient, reducing the quality of health care and leading to increased costs for the organization (3), estimated at 2% of global health expenditure (4).

The practice of healthcare disciplines, the high emotional burden involved in providing care and attention, the characteristics of the health care environment, and the continuous complex decision-making, imply a high exposure to various types of risk factors that jeopardize the mental health of these groups. There is evidence that the risks to the mental health of these groups are observed from their formative stages (5).

As a consequence of the situations experienced by professionals, they may manifest mental health problems such as: anxiety, depression, psychological fatigue, increased substance abuse, sleep and eating disorders, burn-out syndrome, or even suicide (6, 7). This was particularly evident in the impact of the COVID-19 pandemic (8).

From a health promotion approach health and disease as a continuum, where people’s knowledge and skills are tools to exploit their resources and proactively achieve positive health (9–11). Therefore, focusing on available health resources such as Emotional Intelligence, self-care, social support network or development of a sense of coherence, represent a promising way to this effect (12, 13).

Within of these resources, emotional intelligence (EI) was originally defined by Mayer and Salovey (14) as: “the ability to monitor one’s own and others’ feelings and emotions, to discriminate between them, and to use this information to guide one’s thinking and actions.” The same researchers identified the relationship between EI and mental health, where people who develop EI-related skills regulate affect and use moods and emotions to motivate adaptive behavior (15).

In clinical practice, the development of EI skills by healthcare professionals enables them to be more empathetic, resilient and capable of caring for their patients and themselves (16). EI has many implications in the therapeutic relationship, facilitating communication with patients and families experiencing difficult situations due to health problems, and providing emotional support when needed (17–19).

For example, in psychology, nursing and social work students, EI is associated with a lower perceived stress, and the implementation of educational programs on these resources for coping with stress can be developed since their formative stage at university and then transferred to professional practice (20, 21).

On the other hand, a sense of coherence (SOC), is one of the critical elements in promoting health at individual level. It is defined as a global orientation that expresses confidence during the life course when facing stressors from the internal or external environment, in which the person has available resources to cope with such demands (22). A strong SOC helps the person to mobilize resources to cope with stressors and to manage stress successfully (23).

According to the current evidence, SOC is related to different aspects of positive health such as: quality of life, well-being, self-esteem, self-care and healthy lifestyles, among others (24). In university students, SOC is directly related to their mental health (25, 26).

Although the relationship between sense of coherence, emotional intelligence, and health has been demonstrated individually as a health-promoting resource, it is not clear how these variables interact with each other and would provide health benefits to this group.

Given the increased need for mental health promotion interventions among healthcare professionals and students, it would be critical to identify the relationship between sense of coherence and emotional intelligence, how they interact, and how they relate to health and well-being, as well as the potential benefits on other variables are associated with them in students and health professionals.

However, up to our knowledge no available evidence has covered this issue from a in depth review perspective. Therefore, a scoping review was conducted to systematically map the research conducted in this area and to identify existing gaps in knowledge and draw a road map for future research aimed to promote the mental health in this population group.

### 1.1 Aim

The present review seeks to explore the existing literature on the relationship between emotional intelligence and sense of coherence in healthcare workers and students, with the aim of answering the questions: What is the interrelationship between sense of coherence and emotional intelligence, in health professionals since their formative stage? and what are the health benefits driven by this relationship?

## 2 Methods

The review followed the guidelines set out by the Joanna Briggs Institute in “Guidance for conducting systematic scoping reviews” (27) and its subsequent update “Updated methodological guidance for the conduct of scoping reviews” (28). Likewise, the “Preferred Reporting Items for Systematic reviews and Meta-Analyses extension for Scoping Reviews (PRISMA-ScR) Checklist” (29) and the flowchart proposed in “The PRISMA 2020 statement: an updated guideline for reporting systematic reviews” (30) were used.

### 2.1 Search strategy and data sources

From December 15, 2022 to January 10, 2023, a systematic search was conducted using the electronic databases CINAHL (Cumulative Index of Nursing and Allied Health Literature), Latindex, LILACS (Latin American and Caribbean Literature in health Sciences), Psycinfo, Pubmed, Scopus, Science Direct and Web of Science, to identify research that explored emotional intelligence and sense of coherence in university students, as well as health science professionals. The search was updated in January 2024.

Searches were conducted using the following terms ([Students, College Students, University students] OR [Health Personnel, healthcare workers, health professionals, health care settings, professional caregivers] OR [Adults, youth, young people, adolescents]) AND (Emotional Intelligence, emotional competences, emotional education, socio-emotional skills, emotional management, emotional leadership, emotional skills) AND sense of coherence.

The search was constructed based on the first strategy and adapted for the corresponding databases, without the application of additional filters.

### 2.2 Inclusion and exclusion criteria

Basic inclusion criteria were established and adapted as we became more familiar with the literature, so that they were applied to the totality of results obtained (31).

- Types of participants: articles focused on health science university students or professionals were included.
- Concept: the central focus of the articles was emotional intelligence and sense of coherence, as well as their related spheres and concepts.
- Types of articles: primary articles (quantitative and qualitative) and review articles were included. In addition, bibliographic references from leading articles on the topic, as well as from key journals, were reviewed.
- Characteristics of the studies: publications in the last 10 years and available in Spanish or English.

Exclusion criteria included articles on the older adult population, children and participants with special/concrete pathologies.

The selection of the final database of articles was made according to the criteria proposed. One additional article was retrieved by consulting relevant authors and the bibliography of the documents.

### 2.3 Critical appraisal

For the studies selected according to the above criteria, critical appraisal tools will be used to assess the quality of the studies. Depending on the study design, the Joanna Briggs Institute tools (32) will be used, and in the case of mixed methods, the Mixed Methods Assessment Tool (MMAT) (33) will be used. These tools are expected to help assess the reliability, relevance, and results of published articles.

### 2.4 Data extraction

Data extraction was performed following the fields proposed by the Joanna Briggs Institute in “Guidance for conducting systematic scoping reviews” (27).

- Author(s), Year of publication and Country of origin.
- Aim(s) of the study.
- Population and sample (if applicable).
- Study design & Methodology.
- Type of intervention and comparison (if applicable).
- Main results.
- Duration of the intervention (if applicable).
- Measures used.
- Relevant conclusions.

## 3 Results

### 3.1 Descriptive information on studies

**Figure 1:**
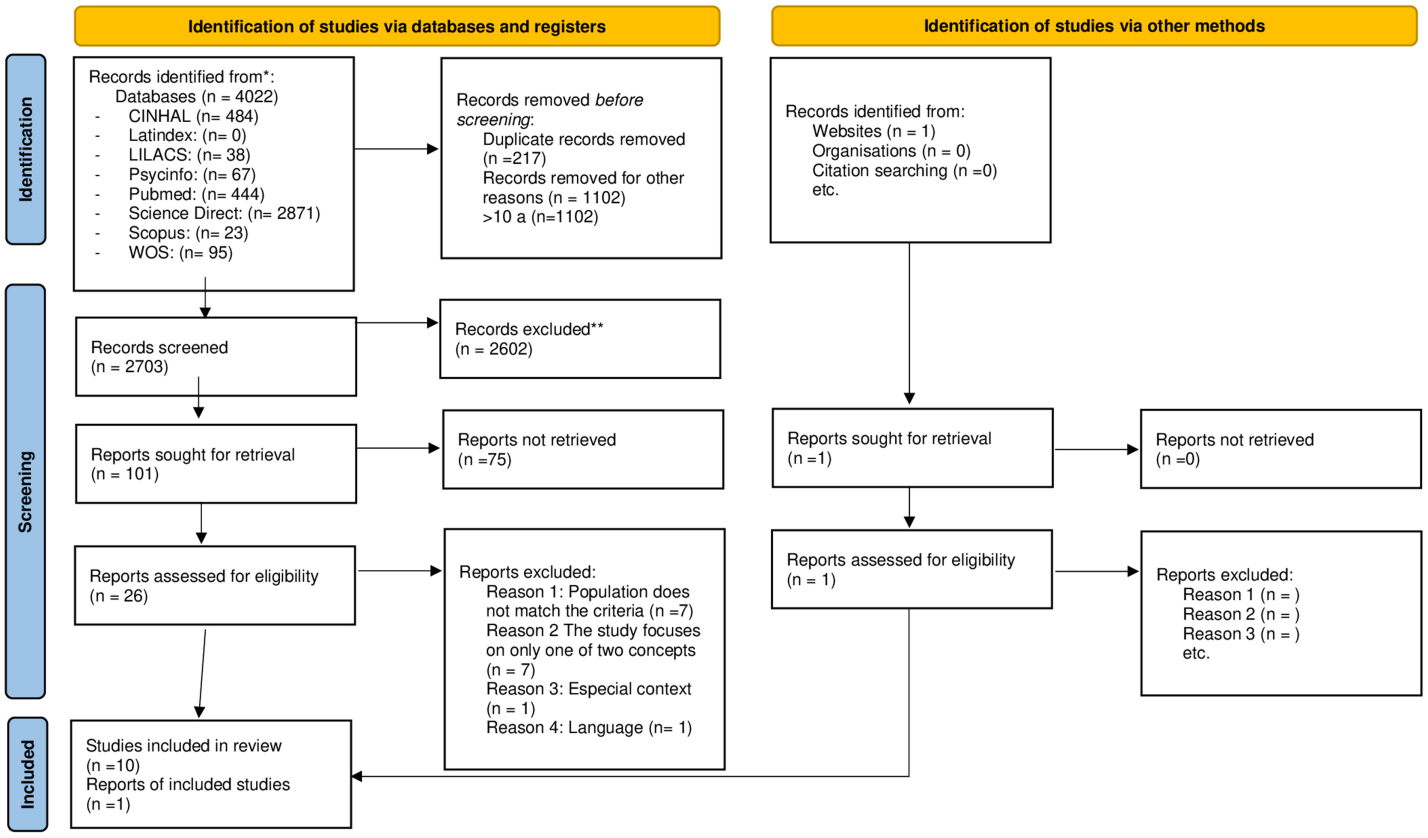
Search strategy and the study selection with PRISMA flow diagram (30).

A total of 4,022 results were identified by searching databases (Figure 1). Once duplicate articles were removed, 2,703 articles were screened by title and abstract. After screening of titles and abstracts, there were 26 studies that met the inclusion criteria for full-text screening, and after full-text reading, 11 studies were included to be reviewed.

The included studies range from the year 2014 to the year 2022. Most of the articles reviewed were original research studies involving primary data collection (91%) (see Table 1). One article used secondary data sets (9%). Most of the studies reviewed used a cross-sectional design (73%). Followed by another mixed-method study (9%) and an intervention study (9%) (see Table 1).

**Table 1:** Description of included studies.

Variable	Number of studies	Percentage of studies (*N* = 11)
**Study type**		
Primary published study	10	91%
Secondary data analysis	1	9%
**Study design**		
Mix method study	1	9%
Cross- sectional	9	82%
Intervention study	1	9%
**Study discipline**		
Nursing	4	37%
Psychology	2	18%
Nursing, psychology	1	9%
Psychology, social science, sociology	2	18%
Nursing, physiotherapy, behavioral science and psychology.	1	9%
Medicine, nursing, psychology, social workers, physiotherapy and dietician.	1	9%
**Country**		
Spain	6	55%
China	2	18%
Poland	1	9%
France	1	9%
Sweden	1	9%

More than half of the studies reviewed were from the fields of nursing or psychology (55%) (see Table 1). A considerable percentage of studies also belonged to the fields of psychology and/or nursing, but included authors or elements from the field of sociology and social science (27%). Finally, a smaller number of studies were interdisciplinary studies from medicine, nursing, as well as physiotherapy, behavioral sciences (9%), or social work and dietetics (9%). The half of the studies came from Spain (55%), followed by China with 2 articles (18%) and other European countries with 1 article each (9%) (see Table 1).

#### 3.1.1 Sample size and description (health professional, student group or mixed group)

The vast majority of the articles had university students as their population (73%). This was followed by articles that only studied health professionals (18%). Only one article (9%) included as study population a mixed group (students and health professionals).

The sample size of the selected articles was, on average, 483 individuals, ranging from 65 to 960. In most studies, women represented more than 80% of the sample; In all samples, men were represented, with an overall mean of less than 20%. The average age of the sample of the studies with a student population was 23.77 years with a range of 16–57 years. For the professional population studies the average was 32.8 years, with a range of 21–60 years. For the mixed population, the sample had an average age of 24 years with a range between 21 and 49 years.

#### 3.1.2 Studies methodological quality assessment

Quality assessment was performed for all 11 papers (Table 2). The Mixed-Methods Appraisal Tool (MMAT) (33) was used for the mixed methods study, and the JBI Critical Appraisal Tools Checklist (32) was used for the cross-sectional studies and the intervention study. Each paper was independently assessed and given an overall rating.

**Table 2:** Quality assessment tool.

Article	1	2	3	4	5	6	7	8	9
JBI cross- sectional									
Fernández-Martínez et al. (2019)	Yes	Unclear	Yes	Yes	Yes	Yes	Yes	Yes	Yes
Colomer-Pérez et al. (2019)	Yes	Unclear	Yes	Yes	Yes	Yes	Yes	Yes	Yes
Burguillos Peña (2014)	Yes	Unclear	No	Yes	Yes	Yes	Yes	Yes	Unclear
Szcześniak and Strochalska (2019)	Yes	Yes	Unclear	Yes	Yes	Yes	Yes	Yes	Unclear
Fernandez-Martinez et al. (2017)	Yes	Yes	Unclear	Yes	Yes	Yes	Yes	Yes	Yes
Shankland et al. (2019)	Yes	Yes	Yes	Yes	Yes	Yes	Yes	Yes	Unclear
Manuel Blanco-Donoso et al. (2018)	Yes	Unclear	Yes	Yes	Yes	Yes	Yes	Yes	Unclear
Hochwälder and Saied (2018)	Yes	Yes	Unclear	Yes	Yes	Yes	Yes	Yes	Yes
Hori et al. (2022)	Yes	Yes	Yes	Yes	Yes	Yes	Yes	Yes	Yes
Article	1	2	3	4	5	6	7	8	9
JBI Interventional study									
Zhan et al. (2020)	Yes	No	No	No	Yes	Unclear	Yes	Yes	Yes
Article	S1	S2	1	2	3	4	5		
Quality Mixed-Methods Appraisal Tool (MMAT)									
Colomer-Pérez et al. (2020)	Yes	Yes	Yes	Yes	Yes	Unclear	Yes		

Furthermore, adequate sampling frame was used by all studies to address the target population. Only 56% of the studies sampled participants properly, the rest were unclear about the sampling or included the entire population. However, 100% of the studies provided a detailed description of their sample and context.

All studies included used appropriate research methods to answer the research question, validated scales to measure the different variables, and used appropriate statistical analysis.

#### 3.1.3 Scales and questionnaires to measure EI and SOC

The sense of coherence was measured using Antonovsky’s (1985) Life Orientation Questionnaire (OLQ) in its original or translated version in 55% of the studies. In the remaining 45% of the studies, the abbreviated version of the questionnaire, either OLQ-13 or SOC-13 (22), was used. Two scales were used to measure emotional intelligence, the Trait Meta- Mood Scale (TMMS) (34) both in its full and abbreviated version, and the emotional intelligence questionnaire (INTE) (35).

Other related dimensions were assessed, such as the level of burnout, which was measured with the MBI-SS (36). Daily hassles were assessed using the Revised Daily Hassles Scale in its French version (37) and the 64-annoyance scale developed by Maybery (38). Emotional exhaustion was assessed using the Emotional Fatigue Scale (ECE) for university students developed by Ramos, Manga, and Moran (39). Other instruments measuring variables other than emotional intelligence and sense of coherence were used in the studies reviewed. These instruments are listed in Table 3. Different strategies were used for the application of the questionnaires. In the vast majority of the studies (64%), the different scales and questionnaires were self-applied presentially. In the remaining studies (36%), online application of the questionnaires was used. The questionnaires were mostly applied in the university setting, and in the hospital setting in those with a professional population.

**Table 3:** Scoping review results.

Author(s), Year of publication and Country of origin	Aim(s) of the study	Population and sample	Study design and methodology	Type of data collection or intervention
Colomer-Pérez et al. (2020) Spain.	To describe the relationship of Health Assets identified in campus. And coping skills in their learning (and living) environment.	921 Students from nursing auxiliary certification (CNA) of all public upper secondary education centers in the Comunitat Valenciana.	Mixed method cross-sectional study. This is a follow-up study of Colomer-Perez et al.’s 2019, conducted in 2016.	Self-administered online questionnaire.
Fernández-Martínez et al. (2019) Spain.	To analyze coping strategies, engagement, EI and SOC and how these variables differentially contribute to self-reported health and well-being.	463 undergraduate nursing students from a Spanish public university.	Cross-sectional study.	Questionnaire application using Lime Survey online tool.
Colomer-Pérez et al. (2019) Spain.	To explore the salutogenic paradigm among nursing assistant students in a region of Spain (Comunitat Valenciana).	First-year Nursing Assistant Certification students from all public upper secondary schools in the Comunitat Valenciana. The sample was 921.	Cross-sectional, analytical and exploratory study conducted in 2016.	Self-administered online questionnaire.
Burguillos Peña (2014) Spain.	The aim of the study was to describe two fundamental variables in people’s health behaviors, a SOC and perceived EI. To analyze the possible cases of social phobia and the tendencies to anxiety and social avoidance. With these, to investigate the relationship that may exist between SOC and social phobia, and between this and perceived EI.	The sample, was composed of 65 students of the Bachelor’s Degree in Psychology and the Diploma in Social Education of the University of Huelva.	Descriptive study.	Application of face-to-face questionnaire.
Szcześniak and Strochalska (2019) Poland.	The research focused on exploring the role of both temperament, as this has been confirmed as a potential component in the development of more complex traits that emerge later in life, and of EI as this has been found to increase SOC.	The sample consisted of 173 participants between 18 and 49 years old.	Cross-sectional study.	Online questionnaire
Fernandez-Martinez et al. (2017) Spain.	To describe the degree of emotional exhaustion and SOC in a sample of university students. In addition, to analyze whether there is a relationship between emotional exhaustion and SOC and this in different academic years and by gender.	Population: University students, sample of 960. The majority are from the field of health sciences.	Cross-sectional study.	Application of the questionnaire in person.
Shankland et al. (2019) France.	The hypothesis was that daily hassles had an effect on academic burnout and that the SOC mediated the relationship between daily hassles and academic burnout. A secondary aim was to explore the relative importance of SOC in explaining academic burnout variance, compared to optimism.	The study was conducted on a sample of 328 third- and fourth-year students in psychology, educational sciences, sports, and sociology in three French universities.	Cross-sectional study.	Questionnaire applied in person.
Manuel Blanco-Donoso et al. (2018) Spain.	The objective of this research is to examine how a particular resource (e.g., cognitive reappraisal) helps people form a SOC.	Psychology students were recruited from Psychology of Personality classes at the Universidad Autónoma de Madrid. The final sample consisted of 214 participants.	Cross-sectional study.	A battery of questionnaires was administered of the variables included in the study.
Zhan et al. (2020) China.	To investigate the mediation role of social support in the relationship between a SOC and the perception of professional interests among Chinese registered nurses.	Population Chinese registered nurses, the sample was 765 nurses.	Cross-sectional study.	Application of a 4-part questionnaire in person.
Hochwälder and Saied (2018) Sweden.	The aim of the study was to test the following two main hypotheses: (1) The avoidance hypothesis: Students with a high SOC experience fewer daily hassles, compared to students with a low SOC. (2) The appraisal hypothesis: Students with a high SOC experience the daily hassles as less stressful, compared to students with a low SOC.	A total of 394 students at a university in Sweden agreed to participate in this study, including students of psychology, economics, education, civil engineering, physiotherapy, nursing and behavioral sciences.	Cross-sectional study.	Application of a questionnaire in person.
Hori et al. (2022) China.	The aims were to test the following three hypotheses: 1. Empathy correlates with self-vigor or self-depression mood in healthcare professionals. 2. Empathy correlates with self-vigor or self-depression mood through SOC mediation. 3. Age moderates the SOC mediation on the relation between empathy and self-moods.	Healthcare professionals (*n* = 132).	Quasi experimental study.	Smile-Sun Method developed by Kazue Takayanagi, consisted in four training sessions applied to different professionals.

Scoping review results, Part 2				
Author(s), Year of publication and Country of origin	Main results	Duration of the intervention	Measures used	Relevant conclusions
Colomer-Pérez et al. (2020) Spain.	The relationship between the intrapersonal HA identified and the scores on the SOC, it is found that those students who outlined aspects related to “caring for others” received higher scores on the SOC than those who identified other introspective behaviors. In the case of the relationship with interpersonal HA, it is found that those who identified their children as health and well-being generating factors have higher SOC scores. Students showed a vocational orientation in choosing this career, they scored higher in SOC and were also the ones who most frequently identified the concept of caring for others within intrapersonal HA.	Time to complete the questionnaire	The questionnaire collected socio demographic data. Some open-ended questions were used to identify HA (intrapersonal, interpersonal, extrapersonal) that enhanced their well-being. SOC levels were assessed using the 13-item Life Orientation Questionnaire (OLQ-13).	These consistent associations between the caregiving factor and the vocational factor with specific reported HA support the salutogenic and asset-based approach. Ultimately, a salutogenic educational strategy rooted in: strengthen CNA students’ SOC, dynamize their HA map, strengthen their sense of vocation, which delves into the zest for healthcare work, consequently enabling them to buffer against work related caregiving stress.
Fernández-Martínez et al. (2019) Spain.	The results show that emotional repair is a mediating variable with little explanatory power when analyzing the relationships between psychological health and SOC. Coping was associated with engagement, EI, and SOC. In addition, nursing students with higher engagement scores also had higher scores for general health, EI, SOC, and some coping strategies. In our sample, psychological health was associated with commitment and vigor of engagement, EI in all its dimensions, SOC, and most of the coping strategies analyzed.	The time required to complete the online questionnaire was not specified by the authors.	The questionnaire used consisted of the following instruments: Students’ health was measured by the Goldberg General Health Questionnaire (GHQ-12). EI was assessed with the Spanish abbreviated version of the Trait Meta-Mood (TMMS-24). Commitment was assessed using the student version of the Utrecht Work Engagement Scale (UWES-Student). SOC was measured using the Sense of Coherence Scale, SOC-13. And coping was measured with the COPE-28 scale.	Those students who perceived better health scored higher on engagement, EI, SOC, and coping strategies. In particular, improving students’ SOC and fostering emotional skills such as repair would help promote the health of individual nursing students. In addition, according to the results of the mediation model, the SOC influences health directly and indirectly through emotional repair.
Colomer-Pérez et al. (2019) Spain.	They found correlations between SOC and being older, being female, and living in an urban environment, factors that appear to influence having a strong SOC, which clearly contributes to having better defense mechanisms against stressors inherent to performance in health sciences.	Time for completion of the questionnaire, not specified by the authors.	A questionnaire developed by the principal investigator was used: SOC assessed by the Life Orientation Questionnaire −13 items (OLQ-13). Subjective mean score to be obtained in the course. Motivation for the choice of studies, measured by developing *ad hoc* categories.	Having a strong SOC seems to contribute to improved stress resistance, which may in part justify the motivation to study something that is enjoyable and to perform well academically, despite being a demanding and stressful profession. This results in professionals who are more resistant to burnout with more internal strategies for professional development, personal satisfaction, and professional competence.
Burguillos Peña (2014) Spain.	The difference in means between the variable “SOC” and “EI” is statistically significant With regard to the relationship between both variables, and the direction of this, we can comment that they have a significant relationship. It is a positive relationship, that is, both score at the same level, the greater the SOC, the greater the EI, and vice versa.	The authors did not specify the time required to complete the questionnaire.	SOC was measured by the SOC-13 questionnaire. EI by TMMS-24, based on the Trait Meta-Mood Scale (TMMS) of the research group of Salovey and Mayer. The Social Anxiety and Avoidance Scale (SAD) was used to assess the social phobia.	Subjects with social phobia (SF) are perceived as having a lower EI than the group of people without phobia. Regarding the SOC, it can be observed that the mean of the clinical cases of SF is lower than the group of “non-cases.” The relationship between SOC and EI is positive, so that both variables would be related to the presence of social phobia.
Szcześniak and Strochalska (2019) Poland.	Based on the obtained results, it can be stated that EI mediates the relationship between temperament, SOC, and global life orientation. The study also showed a negative correlation between depressive temperament and SOC. Also shows positive correlations between hyperthymic temperament, SOC, and EI. Finally, EI acted as a mediator in the relationship between different dimensions of temperament and the SOC.	Time to complete the online questionnaire, not specified by the authors.	Temperament Evaluation of Memphis, Pisa and San Diego Auto questionnaire (TEMPS-A), Orientation to Life Questionnaire (OLQ), and Emotional Intelligence Questionnaire (INTE).	This research provides strong evidence for the mediating role of EI between temperament and SOC. The results support Antonovsky’s theory that individuals’ SOC is largely determined by their resources (temperament) and their ability to perceive, understand, and regulate emotions in the self (EI). In terms of practical implications, the findings suggest the importance of EI training in dealing with less functional temperamental tendencies.
Fernandez-Martinez et al. (2017) Spain.	SOC is negatively related to emotional exhaustion. The results suggest that people who score higher on SOC have lower levels of emotional exhaustion. No differences were found between men and women on the SOC variable, but differences were found on the emotional exhaustion variable, with women scoring higher. Statistically significant differences were observed between the first and second year with regard to the SOC.	The response time was about 20 min.	The abbreviated SOC-13 Sense of Coherence Questionnaire. The Emotional Fatigue Scale (ECE) for university students, by Ramos, Manga and Morán. A questionnaire was used to collect socio-demographic data.	The study confirms the relationship between emotional exhaustion and SOC. There is an inverse relationship between SOC and emotional exhaustion. Differences in SOC were found between first- and third-year students, with higher scores obtained by students in the highest year. This finding would be consistent with Antonovsky’s suggestion that the SOC increases with age up to the age of 30.
Shankland et al. (2019) France.	Direct effects of daily hassles were significant for two dimensions of burnout. SOC was related to the three dimensions of burnout. In addition, all the indirect effects were found to be significant. There was a full mediation of the relationship between daily hassles and the Academic Self-Efficacy subscale by SOC.	Questionnaire completion time, not specified by the authors.	SOC was assessed using the French version of the Sense of Coherence scale (SOC-13). Trait optimism was assessed using the French version of the Life Orientation Test Revised.	As was suggested, SOC mediated the relationship between daily hassles and academic burnout. These results underline the importance of SOC in academic burnout and also suggest that it is important to work on reducing the salience of daily hassles in university students’ minds.
Manuel Blanco-Donoso et al. (2018) Spain.	Cognitive reappraisal and SOC were positively and significantly associated with the variable positive affect. Negative affect correlated significantly and negatively with SOC dimensions, especially with comprehensibility. Examining each SOC dimension, manageability and meaningfulness were significantly and positively associated with cognitive reappraisal.	The authors did not specify the time required to complete the questionnaire.	To measure cognitive appraisal, participants completed the Cognitive Appraisal scale of the Emotional Regulation Questionnaire (ERQ), using the validated and adapted Spanish version of this scale. The Life Orientation Questionnaire (OLQ) was used for SOC. And emotion was measured with the Positive and Negative Affect Scale (PANAS).	The cognitive reappraisal was found to predict positive affect. And also, significantly predicted the manageability, meaningfulness, and usefulness reported by students. The scores obtained on the different dimensions of the SOC were high and predicted positive affect positively and negative affect negatively.
Zhan et al. (2020) China.	SOC was considered as a predictor variable, social support as a mediator variable, and perceived career benefits as an outcome variable. The direct path indicated that a SOC had a direct positive predictive effect on perceived career benefits. The indirect path showed that a SOC, through the function of the mediating variable ‘social support’, had a positive effect on perceived occupational benefits.	Time to complete the 4-part questionnaire, not reported by the authors.	The research questionnaire consisted of four parts: General Information., the SOC Scale (SOC-13), the Social Support Revalued Scale (SSRC), and Perceived Job Benefits Questionnaire.	Social support partially mediates the relationship between a SOC and perceived professional benefits among Chinese registered nurses. Therefore, interventions should be developed based on social support to enhance nurses’ SOC and increase their perceived professional benefits.
Hochwälder and Saied, (2018) Sweden.	Students with a high SOC, compared to students with a low SOC, experienced: fewer daily hassles; daily hassles as less stressful. Furthermore, female and male students did not differ in the number of hassles they experienced, but female students experienced hassles more intensively. There were no significant interactions between SOC and gender on frequency and intensity ratings.	The questionnaire typically took 15–20 min to complete.	After providing their gender, age, and major, participants were asked to provide their responses regarding SOC and daily hassles. SOC was measured using the SOC- 29. Retrospective experience of daily hassles was measured using a scale developed by Maybery.	The results confirm fundamental parts of Antonovsky’s salutogenic model regarding the relationship between SOC and the avoidance and appraisal of stressors in the form of daily hassles. The implications are that efforts should be made to promote higher SOC in students in order to make the effects of daily hassles less harmful.
Hori et al. (2022) China.	The study found that higher levels of empathy were associated with higher SOC, which in turn was positively related to health professionals’ self-efficacy mood. This SOC-mediated relationship was moderated by age. This pattern was also observed with regard to self-depressive mood reduction among health care professionals. A higher empathy was associated with higher SOC, which was then related to lower levels of self-depressed mood.	Training of 4 sessions of one and a half hours each plus the time for the application of the questionnaire.	Empathy was assessed using the Empathy Process Scale (EPS). SOC was assessed with a 13-item abbreviated version of Orientation to Life Questionnaire. Situational mood was assessed with the Profile of Mood States (POMS) short Japanese version.	The results suggest that among healthcare professionals, empathy might promote self-vigor mood and play a protective role against self-depressive mood mediated by SOC. The study suggests the importance of individual differences, such as the growth level of SOC. It can potentially contribute to the design and implementation of interventions to regulate empathy skills, especially for well-being.

Data from the included studies were extracted according to the above-mentioned fields. The results are summarized in Table 3. A summary of the results for each included source of evidence, according to the objectives of the review, is presented below.

### 3.2 Analysis domains found

#### 3.2.1 Predictor and mediator role of emotional intelligence and sense of coherence on health benefits

The sense of coherence (SOC) and emotional intelligence (EI), are considered two variables that influence people’s health (40, 41). SOC is considered a protective factor against stress (41, 42), and a health-promoting resource directly related to the ability to use coping strategies to improve stress management (40).

EI on the other hand, is considered a set of skills that help people to process emotional information, which is developed through learning and experience (40, 43). Both variables are positively related to each other, enhancing their effect, but also mediating between other factors that influence individual well-being (43, 44).

The positive relationship between EI and SOC demonstrates that the development of individual SOC can be determined on the basis of temperament (genetic strengths) and mediated by the emotional resources (EI) of the individual. It is known that EI exerts the mediating role in the development of SOC, this effect may be due to the fact that EI allows monitoring and managing one’s emotions to guide thinking and actions (44).

In the relationship between EI, SOC and health, the direct influence of SOC on health was confirmed, where the improvement of SOC promotes individual health. Likewise, SOC has an indirect influence mediated by EI on health promotion. The mediating role of EI takes effect through emotional repair, which is considered a coping strategy for managing stress associated with problems in university and professional life (40, 41).

The SOC was identified as a mediator between daily stressful events (basis of student and professional stress) (45) and the development of burnout (46). In the role of mediator, SOC has a direct and negative relationship with emotional exhaustion being this a dimension of burnout (47).

The role played by SOC, is positively associated with increased emotional regulation skills (48). Emotional Intelligence interventions appear to be a protective factor, as they lead to an increase in SOC to ultimately benefit individual health (46).

#### 3.2.2 Emotional intelligence training pathway to improve sense of coherence

The implementation of educational strategies related to mental health is mainly based on the salutogenic health model and aims to promote and strengthen the SOC (41, 47). However, according to Shankland et al. (46) it would only make sense to apply these interventions specifically to improve SOC before the age of 30. This would be justified because, according to Antonovsky, SOC as a construct tends to be established after this age, so intervening at earlier stages would be most appropriate.

The use of EI as a training pathway to improve the sense of coherence and health promotion, suggests implementing interventions focused on coping strategies and emotional regulation (45, 46). Increasing emotional regulation tools is considered to be a protective factor that leads to develop a deeper, clearer and more coherent meaning of internal and external challenges or strengthening SOC and that can be intervened through short formative strategies (43, 44, 46, 48).

The proposal arises the need to promote strategies to increase the level of sense of coherence in university students, in order to reduce their emotional exhaustion and improve their health (47). This is due to the fact, that responding successfully to academic and professional stress affects the health of students and future professionals, and is associated with the level of commitment, emotional intelligence and sense of coherence they develop (40).

The intervention selected by Zhan et al. (49) was the Smile-Sun Method developed by Kazue Takayanagi, consisted in four training sessions applied to different professionals from a hospital. They covered elements as laughter theory, practice of drawing natural laughter, and practical laughing training. The objective was to train health professionals on laughter, improving the healing environment through human support, enhancing the natural healing process, motivation and fostering a positive attitude.

As a result of the intervention carried out by the team of Zhan et al. (49), they consider that the young professional population also needs learning opportunities that strengthen their EI, improving their SOC and consequently improving clinical practice. The improvement of SOC seeks to have professionals, who care better and who are also agents of care that implement their tools in the patients they care for (41).

#### 3.2.3 Benefits of SOC and EI on healthcare workers’ mental well-being and engagement

In regard to healthcare professionals, those with a strong SOC are more resistant to burn-out, have more internal professional development strategies, higher personal satisfaction and professional competence, where performance and motivation increase (41, 46, 49). Similarly, clinical performance in professionals is found to be related to EI, associated with high levels of empathy, psychological resilience and life satisfaction (40, 42, 44).

In specific nurse population, it was shown that the relationship between SOC and professional satisfaction is mediated by social support, and that social support has a direct predictive effect on perceived professional benefits, which acts as an internal motivator increasing individual well-being (49).

Within the same line Colomer-Pérez et al. (41) and Hochwälder and Saied (45), identified that in university students a strong SOC seems to contribute to greater resilience to stress, which would justify the motivation to study a profession with high demands and stressors. In students, the perception of a high level of health is associated with higher levels of engagement, EI, SOC and coping strategies (40).

According to Colomer-Pérez et al. (50) the use of a salutogenic educational strategy whose objective is to strengthen students’ SOC, allows them to dynamize their asset maps, reinforcing their sense of vocation, allowing professionals to fight against care stress and thrive in their profession.

## 4 Discussion

To our knowledge, this scoping review represents a first attempt to describe the literature on the association between SOC and EI and the potential mental health benefits of these mutual health assets in students and professionals from different health disciplines. Several key findings can be highlighted from the review.

First, this review found evidence that demonstrates the relationship between a sense of coherence and emotional intelligence, where the relationship between both variables is fundamental in health promotion. Each variable plays an important role, acting both as a mediator and as a predictor (44). Second, evidence suggests that using EI represents a key training pathway to improve SOC and health outcomes. EI is associated with the response to academic and clinical stress that affects healthcare students (45, 46). Likewise, it is identified that EI plays an important role in the training of students, for the development of skills and the strengthening of the SOC early on, seeking to obtain professionals with better tools for the management of stressful situations (51).

Third, the benefits obtained by healthcare professionals and students by intervening on EI and SOC, contribute to improve general well-being, commitment and motivation for a better performance in studies and clinical work (52). Consistent with the results, recent reviews highlight that EI and interventions on this variable have the benefit of improving productivity and clinical performance (53, 54).

The present review also highlights the existence of gaps and limitations in the literature. First, it shows the existence of studies, although few, on the relationship between SOC and EI. At the same time, it highlights the lack of studies that allow us to identify casual relationships of intervening in any or both of these variables to promote mental health in healthcare sector.

### 4.1 Limitations of the scoping review

This review has several limitations. Due to the heterogeneity of the results and despite the homogeneity of the study designs, we could only summarize the results narratively. And we could not aggregate the results regarding the association between SOC, EI, and mental health using meta-analytic techniques. However, this was not the aim of our review.

Furthermore, the exploratory nature of the scoping review (55) allowed us to capture a diverse range of evidence, thereby providing a more complete overview of the state of the literature to date. The current evidence on the subject is mostly focused on one of the two variables and its possible benefits on health care professionals’ health outcomes. More than half of the studies found have a cross-sectional (82%) design, which does not allow us to evaluate the effect on either variable or to identify changes and changing mechanisms. At the same time, the proportion of women in all the studies is over 65%, which would not allow us to identify significant gender differences in the variables studied.

With regard to the results, in all the studies they may be biased by the sampling method, since the sample of participants is limited to a single university or hospital, which may jeopardize the validity of the data obtained, leaving open the possibility that the participants are not representative of students and health professionals in general (42).

It should be noted that most of the samples in the studies included only one or two healthcare disciplines, such as nursing, social work, or psychology. Some studies included mixed samples of health science students. Therefore, it was not possible to make a direct comparison between these studies. Furthermore, future research with mixed samples of healthcare workers is needed to ensure representativeness. The available evidence is currently limited to specific samples, which may not be representative of health worker groups as a whole. To overcome these limitations, it is suggested that future research should use longitudinal and analytical studies to investigate the indirect and direct relationship between SOC and EI and their possible associated variables when exploring the impact of mental health promotion interventions in this sector.

It is also suggested that studies be conducted with mixed populations, as the current evidence focuses mainly on students or professionals. Likewise, it is suggested that the population be expanded to include different healthcare professions, since most of the evidence currently focuses on nursing and psychology, leaving out other professions that work in the healthcare context.

Similarly, the review assessed the methodological quality of each included study, but did not assess the risk of within-study bias, as this is generally considered less applicable to scoping reviews (29).

The current review included only quantitative evidence due to a lack of qualitative evidence on the topic. However, it is likely that qualitative data will provide a more nuanced picture of the relationship between SOC, EI and mental health, as well as important information about coping mechanisms and the context in which stress is experienced and managed.

The decision to limit the inclusion of articles and focus only on those that specifically used the terms SOC and EI with some related variables may have resulted in the omission of relevant literature on similar constructs that used different terminology.

### 4.2 Theoretical and practical implications

The present review aims to stimulate research-action-prevention aimed at promoting the mental health of students and health professionals. Although working conditions generally expose professionals to stressful situations, an increase in psychological distress, as was observed during the COVID-19 pandemic, it is now well-known that health workers’ sense of coherence and their EI skills can serve as critical individual protectors for health risks (56, 57). To ensure that healthcare professionals can effectively manage the effects of working in stressful circumstances, it is considered important to intervene to protect their mental health (58).

This becomes even more necessary in light of the limited evidence on psychosocial interventions aimed at strengthening EI and SOC. Interventions on EI based on the salutogenic approach during university studies are considered to strengthen the sense of coherence and thus promote their health (50). According to Fragkos and Crampton (59), interventions to develop empathy in a specific population of medical students are effective in those under 30 years of age.

However, the effectiveness of an educational program applied to students of other health professions has not been clearly demonstrated (60, 61). Therefore, it would be advisable to study this topic in depth in order to find the most effective methodology and educational program.

Regarding interventions for health professionals, the current evidence is considered uncertain, as the effectiveness of one intervention over another in reducing stress, increasing resilience, and improving health has not been demonstrated (62–64). According to Pollock et al. (65), there is a lack of quantitative and qualitative data from studies conducted during or after epidemics and pandemics that can inform the selection of interventions beneficial to the mental health of healthcare professionals.

## 5 Conclusion

This scoping review sought to explore the relationship between emotional intelligence and sense of coherence in healthcare professionals and students, to clarify the interrelationship between the two and how they influence their health.

The results of this review suggest that both factors influence health and that the relationship between them allows them to act as either mediators or predictors, both of which are fundamental for health promotion in health professionals and students. The data from the scoping review suggest that in order to intervene, it is necessary to use EI as a training pathway to improve SOC and thus impacting on other health outcomes. It also suggested that this mental health interventions should be done early in university students and young health professionals.

This study may highlight the need to intervene in EI to improve the well-being and engagement of students, as well as the clinical performance in their professional stage. However, it also highlights the lack of literature on this topic and the need for evidence of interventions’ efficacy and effectiveness.

### 5.1 Relevance for clinical practice

The purpose of this review was to identify and synthesize the available evidence on the relationship between these variables. By identifying the benefits of the relationship between these variables and the best way to intervene, we could lay the foundations for the design, implementation and evaluation plan of future mental health interventions in this population.

Intervening on these variables would allow the promotion of mental health and well-being in healthcare professionals from their early formative stage. It will allow them to develop mental health protectors’ factors, adopting effective coping skills to manage stress and reducing physical health risks in the context of work and study.

## Author contributions

VU-H: Conceptualization, Data curation, Formal analysis, Investigation, Methodology, Project administration, Resources, Validation, Visualization, Writing – original draft, Writing – review & editing. MN-S: Conceptualization, Data curation, Formal analysis, Investigation, Methodology, Resources, Supervision, Validation, Visualization, Writing – original draft, Writing – review & editing, Project administration. AP-S: Conceptualization, Data curation, Funding acquisition, Investigation, Methodology, Project administration, Resources, Supervision, Validation, Writing – review & editing. AP-M: Data curation, Investigation, Methodology, Supervision, Validation, Visualization, Writing – review & editing. AG: Conceptualization, Data curation, Investigation, Methodology, Project administration, Resources, Software, Writing – original draft. EL: Funding acquisition, Investigation, Methodology, Project administration, Resources, Supervision, Validation, Visualization, Writing – review & editing. EB-M: Conceptualization, Data curation, Formal analysis, Investigation, Methodology, Project administration, Resources, Supervision, Validation, Visualization, Writing – original draft, Writing – review & editing, Funding acquisition.

## References

[1] World Health Organization. WHO guidelines on mental health at work. Geneva: World Health Organization (2022).

[2] World Health Organization. Comprehensive mental health action plan 2013–2030. Geneva: World Health Organization (2021).

[3] De Simone S. Wellbeing at work: a survey on perception of health care workers. Riv Int Sci Sociali. (2015) 123:395–412.

[4] World Health Organization. Occupational health: health workers. Geneva: World Health Organization (2022).

[5] Snowden A Stenhouse R Duers L Marshall S Carver F Brown N. The relationship between emotional intelligence, previous caring experience and successful completion of a pre-registration nursing/midwifery degree. J Adv Nurs. (2018) 74:433–42. doi: 10.1111/jan.13455, PMID: 28910494

[6] Serrano-Ripoll MJ Meneses-Echavez JF Ricci-Cabello I Fraile-Navarro D Fiol-deRoque MA Pastor-Moreno G. Impact of viral epidemic outbreaks on mental health of healthcare workers: a rapid systematic review and meta-analysis. J Affect Disord. (2020) 277:347–57. doi: 10.1016/j.jad.2020.08.034, PMID: 32861835 PMC7443314

[7] Dosil Santamaría M Ozamiz-Etxebarria N Redondo Rodríguez I Jaureguizar Alboniga-Mayor J Picaza GM. Psychological impact of COVID-19 on a sample of Spanish health professionals. Psychiatry Ment Heal J. (2021) 14:106–12. doi: 10.1016/j.rpsmen.2020.05.002 PMC7264016 32622882

[8] Greenberg N Docherty M Gnanapragasam S Wessely S. Managing mental health challenges faced by healthcare workers during COVID-19 pandemic. BMJ. (2020) 368:m1211. doi: 10.1136/bmj.m1211 32217624

[9] Antonovsky A. The salutogenic model as a theory to guide health promotion. Health Promot Int. (1996) 11:11–8. doi: 10.1093/heapro/11.1.11

[10] Golembiewski JA. Salutogenic architecture in healthcare settings In: Mittelmark MB Sagy S Eriksson M Bauer GF Pelikan JM Lindström B, editors. The Handbook of Salutogenesi. Cham: Springer (2017). 267–76. 28590652

[11] Bauer GF Roy M Bakibinga P Contu P Downe S Eriksson M. Future directions for the concept of salutogenesis: a position article. Health Promot Int. (2020) 35:187–95. doi: 10.1093/heapro/daz057, PMID: 31219568

[12] Bermejo-Martins E Luis EO Fernández-Berrocal P Martínez M Sarrionandia A. The role of emotional intelligence and self-care in the stress perception during COVID-19 outbreak: an intercultural moderated mediation analysis. Pers Individ Dif. (2021) 177:110679. doi: 10.1016/j.paid.2021.110679, PMID: 36540668 PMC9756564

[13] Luis E Bermejo-Martins E Martinez M Sarrionandia A Cortes C Oliveros EY. Relationship between self-care activities, stress and well-being during COVID-19 lockdown: a cross-cultural mediation model. BMJ Open. (2021) 11:e048469. doi: 10.1136/bmjopen-2020-048469, PMID: 34911708 PMC8678542

[14] Salovey P Mayer JD. Emotional intelligence. Imagin Cogn Pers. (1989) 9:185–211. doi: 10.2190/DUGG-P24E-52WK-6CDG

[15] Mestre JM Brackett MA Guil R Salovey P. Emotional intelligence: definition, assessment and applications from Mayer and Salovey’s skills model In: Motivation and emotion. New York: McGraw-Hill USA (2008). 407–38.

[16] Nightingale S Spiby H Sheen K Slade P. The impact of emotional intelligence in health care professionals on caring behaviour towards patients in clinical and long-term care settings: findings from an integrative review. Int J Nurs Stud. (2018) 80:106–17. doi: 10.1016/j.ijnurstu.2018.01.006, PMID: 29407344

[17] Mayer JD. What is emotional intelligence?. 8, University New Hamshire Personality Lab. New Hampshire; (2004). 1–10

[18] Mayer JD Salovey P Caruso DR. TARGET ARTICLES: “Emotional intelligence: theory, findings, and implications”. Psychol Inq. (2009) 2014:37–41. doi: 10.1207/s15327965pli1503_02

[19] Hajibabaee F Farahani MA Ameri Z Salehi T Hosseini F Ameri Z. The relationship between empathy and emotional intelligence among Iranian nursing students. Int J Med Educ. (2018) 9:239–43. doi: 10.5116/ijme.5b83.e2a5, PMID: 30244237 PMC6387768

[20] Cobb CD Mayer JD. Emotional intelligence. Educ Leadersh. (2000) 58:14–8. doi: 10.2190/dugg-p24e-52wk-6cdg

[21] Enns A Eldridge GD Montgomery C Gonzalez VM. Perceived stress, coping strategies, and emotional intelligence: a cross-sectional study of university students in helping disciplines. Nurse Educ Today. (2018) 68:226–31. doi: 10.1016/j.nedt.2018.06.012, PMID: 30053557

[22] Mittelmark MB Sagy S Eriksson M Bauer GF Pelikan JM Lindström B. The sense of coherence and its measurement In: Eriksson M Mittelmark MB, editors. The handbook of Salutogenesis. Cham: Springer (2017). 97–106. 28590637

[23] Moksnes UK. Sense of coherence In: Haugan G Eriksson M, editors. Health promotion in health care – vital theories and research. Cham: Springer (2021). 35–46. 36315659

[24] Vega Martínez MC d Frías Osuna A del Pino Casado R. Validity and reliability of the sense of coherence scale among nursing undergraduate students from a Spanish university. Gac Sanit. (2019) 33:310–6. doi: 10.1016/j.gaceta.2018.02.009 30078500

[25] Mato M Tsukasaki K. Factors promoting sense of coherence among university students in urban areas of Japan: individual-level social capital, self-efficacy, and mental health. Glob Health Promot. (2019) 26:60–8. doi: 10.1177/1757975917691925 28382845

[26] Eriksson M Lindström B. Antonovsky’s sense of coherence scale and the relation with health: a systematic review. J Epidemiol Commun Health. (2006) 60:376–81. doi: 10.1136/jech.2005.041616, PMID: 16614325 PMC2563977

[27] Peters MDJ Godfrey CM Khalil H McInerney P Parker D Soares CB. Guidance for conducting systematic scoping reviews. Int J Evid Based Heal. (2015) 13:141–6. doi: 10.1097/XEB.0000000000000050 26134548

[28] Peters MDJ Marnie C Tricco AC Pollock D Munn Z Alexander L. Updated methodological guidance for the conduct of scoping reviews. JBI Evid Synth. (2020) 18:2119–26. doi: 10.11124/JBIES-20-00167 33038124

[29] Tricco AC Lillie E Zarin W O’Brien KK Colquhoun H Levac D. PRISMA extension for scoping reviews (PRISMA-ScR): checklist and explanation. Ann Intern Med. (2018) 169:467–73. doi: 10.7326/M18-0850, PMID: 30178033

[30] Page MJ McKenzie JE Bossuyt PM Boutron I Hoffmann TC Mulrow CD. The PRISMA 2020 statement: an updated guideline for reporting systematic reviews. BMJ. (2021) 372:n71. doi: 10.1136/bmj.n71, PMID: 33782057 PMC8005924

[31] Arksey H O’Malley L. Scoping studies: towards a methodological framework. Int J Soc Res Methodol. (2005) 8:19–32. doi: 10.1080/1364557032000119616

[32] Joanna Briggs Institute. Critical appraisal tools. JBI Manual for Evidence Synthesis. (2020). Available at: https://jbi.global/critical-appraisal-tools (Accessed April 19, 2023).

[33] Hong QN Fàbregues S Bartlett G Boardman F Cargo M Dagenais P. The mixed methods appraisal tool (MMAT) version 2018 for information professionals and researchers. Educ Inf. (2018) 34:285–91. doi: 10.3233/EFI-180221

[34] Salovey P Mayer JD Goldman SL Turvey C Palfai TP. Emotional attention, clarity, and repair: exploring emotional intelligence using the trait meta-mood scale. In Pennebaker J. W. (Ed.) Emotion, disclosure, and health. Washington, DC, US: American Psychological Association; (1995). p. 125–154.

[35] Schutte NS Malouff JM Hall LE Haggerty DJ Cooper JT Golden CJ. Development and validation of a measure of emotional intelligence. Pers Individ Dif. (1998) 25:167–77. doi: 10.1016/S0191-8869(98)00001-4

[36] Schaufeli WB Martínez IM Pinto AM Salanova M Barker AB. Burnout and engagement in university students a cross-national study. J Cross-Cult Psychol. (2002) 33:464–81. doi: 10.1177/0022022102033005003

[37] Holm JE Holroyd KA. The daily hassles scale (revised): does it measure stress or symptoms? Behav Assess. (1992) 14:465–82.

[38] Maybery D. Incorporating interpersonal events within hassle measurement. Stress Heal. (2003) 2:97–110. doi: 10.1002/smi.961

[39] Moran Astorga C Ramos Campos F Manga RD. Escala de cansancio emocional (ECE) para estudiantes universitarios. Interpsiquis. (2005) 6:1–9

[40] Fernández-Martínez E López-Alonso AI Marqués-Sánchez P Martínez-Fernández MC Sánchez-Valdeón L Liébana-Presa C. Emotional intelligence, sense of coherence, engagement and coping: a cross-sectional study of university students’ health. Sustain For. (2019) 11:6953. doi: 10.3390/su11246953

[41] Colomer-Pérez N Paredes-Carbonell JJ Sarabia-Cobo C Gea-Caballero V. Sense of coherence, academic performance and professional vocation in certified nursing assistant students. Nurse Educ Today. (2019) 79:8–13. doi: 10.1016/j.nedt.2019.05.004, PMID: 31078870

[42] Hori M Yoshikawa E Hayama D Sakamoto S Okada T Sakai Y. Sense of coherence as a mediator in the association between empathy and moods in healthcare professionals: the moderating effect of age. Front Psychol. (2022) 13:13. doi: 10.3389/fpsyg.2022.847381 PMC9083206 35548503

[43] Burguillos Peña AI. Sense of coherence and emotional intelligence: effect on social anxiety of college students. Int J Dev Educ Psychol. (2014) 24:45–54. doi: 10.17060/ijodaep.2014.n1.v4.615

[44] Szcześniak M Strochalska K. Temperament and sense of coherence: emotional intelligence as a mediator. Int J Environ Res Public Health. (2019) 17:219. doi: 10.3390/ijerph17010219, PMID: 31892262 PMC6981951

[45] Hochwälder J Saied V. The relation between sense of coherence and daily hassles among university students. Heal Psychol Behav Med. (2018) 6:329–39. doi: 10.1080/21642850.2018.1538802, PMID: 34040835 PMC8114344

[46] Shankland R Kotsou I Vallet F Bouteyre E Dantzer C Leys C. Burnout in university students: the mediating role of sense of coherence on the relationship between daily hassles and burnout. High Educ. (2019) 78:91–113. doi: 10.1007/s10734-018-0332-4

[47] Fernández-Martinez E Liebana-Presa C Moran AC. Relationship between sense of coherence and emotional exhaustion in university students. Psychol Soc Educ. (2017) 9:393–403. doi: 10.25115/psye.v9i3.861

[48] Manuel Blanco-Donoso L Moreno-Jimenez B Boada M Rodriguez-Carvajal R Garrosa E. Emotional regulation and positive affect: the mediator effect of sense of coherence. Rev Argentina Clínica Psicológica. (2018) 27:403–12. doi: 10.24205/03276716.2018.1053

[49] Zhan T Li H Ding X. Can social support enhance sense of coherence and perceived professional benefits among Chinese registered nurses? A mediation model. J Nurs Manag. (2020) 28:488–94. doi: 10.1111/jonm.12931, PMID: 31845402

[50] Colomer-Pérez N Chover-Sierra E Gea-Caballero V Paredes-Carbonell JJ. Health assets, vocation and zest for healthcare work. A Salutogenic approach to active coping among certified nursing assistant students. Int J Environ Res Public Health. (2020) 17:3586. doi: 10.3390/ijerph17103586, PMID: 32443778 PMC7277762

[51] Cleary M Visentin D West S Lopez V Kornhaber R. Promoting emotional intelligence and resilience in undergraduate nursing students: an integrative review. Nurse Educ Today. (2018) 68:112–20. doi: 10.1016/j.nedt.2018.05.018, PMID: 29902740

[52] Hernández-Vargas CI Llorens-Gumbau S Rodríguez-Sánchez AM Chambel MJ. Emotional intelligence and engagement in medicine students: a comparative study in three countries. Rev Psicol. (2021) 30:44–56. doi: 10.5354/0719-0581.2021.55261

[53] Singh N Kulkarni S Gupta R. Is emotional intelligence related to objective parameters of academic performance in medical, dental, and nursing students: a systematic review. Educ Heal Chang Learn Pract. (2020) 33:8–12. doi: 10.4103/efh.EfH_208_17 32859874

[54] Dugué M Sirost O Dosseville F. A literature review of emotional intelligence and nursing education. Nurse Educ Pract. (2021) 54:103124. doi: 10.1016/j.nepr.2021.103124 34175653

[55] Tricco AC Lillie E Zarin W O’Brien K Colquhoun H Kastner M. A scoping review on the conduct and reporting of scoping reviews. BMC Med Res Methodol. (2016) 16:15–12. doi: 10.1186/s12874-016-0116-4, PMID: 26857112 PMC4746911

[56] Gomez-Salgado J Dominguez-Salas S Romero-Martin M Ortega-Moreno M Jesus Garcia-Iglesias J Ruiz-Frutos C. Sense of coherence and psychological distress among healthcare workers during the COVID-19 pandemic in Spain. Sustain For. (2020) 12:6855. doi: 10.3390/su12176855

[57] Gómez-Salgado J Ortega-Moreno M Soriano G Fagundo-Rivera J Allande-Cussó R Ruiz-Frutos C. History of contact with the SARS-COV-2 virus and the sense of coherence in the development of psychological distress in the occupational health professionals in Spain. Sci Prog. (2021) 104:368504211026121. doi: 10.1177/00368504211026121, PMID: 34137643 PMC10454955

[58] Gómez-Salgado J Arias-Ulloa CA Ortega-Moreno M García-Iglesias JJ Escobar-Segovia K Ruiz-Frutos C. Sense of coherence in healthcare workers during the COVID-19 pandemic in Ecuador: association with work engagement, work environment and psychological distress factors. Int J Public Health. (2022) 67:1605428. doi: 10.3389/ijph.2022.1605428, PMID: 36545403 PMC9760665

[59] Fragkos KC Crampton PES. The effectiveness of teaching clinical empathy to medical students: a systematic review and meta-analysis of randomized controlled trials. Acad Med. (2020) 95:947–57. doi: 10.1097/ACM.0000000000003058, PMID: 31688037

[60] Everson N Levett-Jones T Pitt V. The impact of educational interventions on the empathic concern of health professional students: a literature review. Nurse Educ Pract. (2018) 31:104–11. doi: 10.1016/j.nepr.2018.05.015, PMID: 29852474

[61] Cleary M Kornhaber R Thapa DK West S Visentin D. The effectiveness of interventions to improve resilience among health professionals: a systematic review. Nurse Educ Today. (2018) 71:247–63. doi: 10.1016/j.nedt.2018.10.002 30342300

[62] Kunzler AM Helmreich I Chmitorz A König J Binder H Wessa M. Psychological interventions to foster resilience in healthcare professionals. Cochrane Database Syst Rev. (2020) 2020:CD012527. doi: 10.1002/14651858.CD012527.pub2, PMID: 32627860 PMC8121081

[63] Tement S Ketiš ZK Miroševič Š Selič-Zupančič P. The impact of psychological interventions with elements of mindfulness (PIM) on empathy, well-being, and reduction of burnout in physicians: a systematic review. Int J Environ Res Public Health. (2021) 18:1–12. doi: 10.3390/ijerph182111181 PMC8582910 34769700

[64] Venegas CL Nkangu MN Duffy MC Fergusson DA Spilg EG. Correction: interventions to improve resilience in physicians who have completed training: a systematic review. PLoS One. (2019) 14:e0210512. doi: 10.1371/journal.pone.0210512 30653550 PMC6336384

[65] Pollock A Campbell P Cheyne J Cowie J Davis B McCallum J. Interventions to support the resilience and mental health of frontline health and social care professionals during and after a disease outbreak, epidemic or pandemic: a mixed methods systematic review. Cochrane Database Syst Rev. (2020) 2020:CD013779. doi: 10.1002/14651858.CD013779, PMID: 33150970 PMC8226433

